# Hemodiafiltration: Technical and Medical Insights

**DOI:** 10.3390/bioengineering10020145

**Published:** 2023-01-21

**Authors:** Thomas Lang, Adam M. Zawada, Lukas Theis, Jennifer Braun, Bertram Ottillinger, Pascal Kopperschmidt, Alfred Gagel, Peter Kotanko, Manuela Stauss-Grabo, James P. Kennedy, Bernard Canaud

**Affiliations:** 1 Global Biomedical Evidence Generation, Fresenius Medical Care Deutschland GmbH, 61352 Bad Homburg, Germany; 2 Product Development, Fresenius Medical Care Deutschland GmbH, 66606 Sankt Wendel, Germany; 3 Ottillinger Life Sciences, 85649 Brunnthal, Germany; 4 Hemodialysis Machines Germany Innovative Technology, Fresenius Medical Care Deutschland GmbH, 97424 Schweinfurt, Germany; 5 Hemodialysis Machines Global Systems Engineering, Fresenius Medical Care Deutschland GmbH, 97424 Schweinfurt, Germany; 6 Renal Research Institute, New York, NY 10065, USA; 7 Icahn School of Medicine at Mount Sinai, New York, NY 10029, USA; 8 School of Medicine, Montpellier University, 34090 Montpellier, France; 9 Global Medical Office, Fresenius Medical Care Deutschland GmbH, 61352 Bad Homburg, Germany

**Keywords:** hemodiafiltration, performance, convection volume, end-stage kidney disease, dialysis

## Abstract

Despite the significant medical and technical improvements in the field of dialytic renal replacement modalities, morbidity and mortality are excessively high among patients with end-stage kidney disease, and most interventional studies yielded disappointing results. Hemodiafiltration, a dialysis method that was implemented in clinics many years ago and that combines the two main principles of hemodialysis and hemofiltration—diffusion and convection—has had a positive impact on mortality rates, especially when delivered in a high-volume mode as a surrogate for a high convective dose. The achievement of high substitution volumes during dialysis treatments does not only depend on patient characteristics but also on the dialyzer (membrane) and the adequately equipped hemodiafiltration machine. The present review article summarizes the technical aspects of online hemodiafiltration and discusses present and ongoing clinical studies with regards to hard clinical and patient-reported outcomes.

## 1. Introduction

Patients with end-stage kidney disease (ESKD) are a severely ill population with a complex comorbidity situation and high mortality rates [1,2,3,4,5,6]. There is a clear need to improve these hard clinical endpoints and the quality of life of ESKD patients. Most of the patients depend on an extracorporeal renal replacement therapy, such as low- and high-flux hemodialysis (HD) or hemodiafiltration (HDF). HDF is in widespread use, especially in Europe and Asia, but less so in the United States; here, fewer HDF systems have been cleared by the Food and Drug Administration (FDA) [7,8,9].

HDF combines the diffusion of mainly low molecular weight uremic toxins, known from conventional HD, with the convection of soluble middle-sized toxins, such as β2-microglobulin, within the same high-flux hemodialyzer module [7,10,11]. HDF is considered the most advanced renal replacement therapy that is currently available [7], as clinical studies have demonstrated its superiority in removing middle- and large-sized uremic toxins as compared to HD. Moreover, HDF offers the potential to improve hard clinical outcomes, and clinical studies have shown very promising results [12,13,14,15,16].

Firstly, the present review article describes the technical considerations of HDF with regards to the dialyzer and the machine to achieve the best potential of HDF. Secondly, medical considerations of HDF are discussed in this article, which summarizes the current clinical evidence on performance and hard clinical endpoints. Finally, the review article provides an outlook on ongoing clinical trials on HDF investigating hard clinical endpoints and patient-reported outcomes [17,18].

## 2. Technical Insights of HDF

HDF dates back to the late 1960s and has been improved continuously thereafter [19]. It is a form of kidney replacement therapy that combines the principles of HD and hemofiltration (HF) [20]. In conventional HD, the solute removal is primarily achieved via diffusion, which is the movement of molecules along a concentration gradient between the blood and dialysate that is higher for small molecules. In contrast, solute removal in HF is based on convective transport, which depends on the ultrafiltration rate and is equal for different molecule sizes as long as they can pass through the pores of the membrane, reflecting its sieving capacity. In HDF, diffusive and convective mechanisms are combined, resulting in the high removal of small molecules while also obtaining a high removal of larger molecules. However, as diffusive and convective transports are affecting each other, the total clearance by the combination of both techniques is not as high as the sum of the clearances of each single technique alone; diffusion lowers a solute’s concentration in the blood and, thereby, the potential removal capacity via convective transport. Vice versa, a solute’s removal via convection lowers the concentration gradient between the blood and dialysate and, thereby, reduces the potential removal capacity via diffusive transport [21,22].

The following three major technological components are required to perform HDF: a substitution fluid, a hemodiafilter, and an HDF machine. The following sections review technical insights for each of these three major components and further address how synergy between the hemodiafilter and the HDF machine can help maximize the substitution volume.

### 2.1. The Substitution Fluid

HDF removes high plasma water volumes via ultrafiltration, which, in turn, needs to be replaced isovolumetrically with a substitution fluid. This substitute fluid is infused into the blood of the patient and, therefore, needs to be sterile and non-pyrogenic. There are several modes of replacement therapies available. Of these, the following are described in more detail: post-dilution, pre-dilution, mixed-dilution, and mid-dilution HDF [21].

Post-dilution HDF: The substitution fluid is infused downstream of the dialyzer into the venous side of the extracorporeal circuit. Post-dilution HDF offers high convective clearances and removal rates of soluble uremic toxins at normal or higher blood flow rates. The high ultrafiltration rate results in an increase in the serum protein concentrations due to the high water removal and, thereby, an increase in blood viscosity and oncotic pressure, which, in turn, can lead to membrane fouling (see also Section 2.4) [11,22]. Post-dilution is the most commonly used mode of online HDF [11]. These factors limit the filtration fraction to around 30% of the blood flow rate [22].

Pre-dilution HDF: The substitution fluid is infused upstream of the dialyzer into the arterial side of the extracorporeal circuit. In the pre-dilution mode, the solute concentrations in the blood are reduced, resulting in lower diffusive and convective clearance rates compared with the post-dilution mode [22]. Pre-dilution HDF decreases the hematocrit and oncotic pressure while preserving the transmembrane pressure gradient along the capillaries, reducing the risk of clot formation and shear stress inside the capillaries; thus, it may reduce the formation of a “secondary membrane” as a polarization of proteins on the dialyzer’s inner membrane surface [23]. This may be especially important if the membrane is prone to such accumulation, as secondary membranes change a dialyzer’s performance and characteristics. It facilitates superior convective clearances in some particular clinical conditions associated with low blood flow regimens (i.e., children, low access flow, and central venous catheters) or unfavorable hemorheological conditions (i.e., high protein concentration and high hematocrit) [23,24,25,26,27,28,29]. It requires a larger (twice as large) substitution volume to achieve equivalent solute clearances as in post-dilution HDF since it dilutes solutes entering the hemodialyzer. Pre-dilution HDF is commonly used in Japan, where traditionally applied low blood flow rates favor this modality.

Mixed-dilution HDF: In mixed-dilution HDF, the substitution fluid is infused simultaneously at different rates (typically 80/20%) before and after the dialyzer, avoiding some of the shortcomings of pre- and post-dilution HDF. It, however, requires specific blood tubing and a non-standard dialysis machine with an additional pump.

Mid-dilution HDF: Mid-dilution HDF is a non-conventional modality that requires a special dialyzer with a specific inlet port for the replacement fluid, allowing for pre- and post-dilution. The housing of this dialyzer contains two high-flux fiber bundles—an outer annular region and an inner core region—divided by a special header cap. Firstly, the blood is passed through the annular outer bundle in the post-dilution mode and is then mixed with the substitution fluid at the opposite end of the dialyzer. Secondly, the diluted blood is passed through the inner core of the dialyzer in the reverse direction to the dialyzer blood exit in the pre-dilution mode [30,31].

Of note, several factors during HDF treatments affect each other, including blood flow or convective volume, and are important determinants among the different substitution modalities to achieve the respective treatment goals. For example, in certain conditions where low blood flow is necessary, the convective volume target may nonetheless be achieved. Here, the dilution factor of the respective HDF mode plays the following important role: when compared to post-dilution HDF, the dilution factor for pre-dilution HDF is 2, 1.5 for mixed-dilution HDF, and also 1.5 for mid-dilution HDF. Thus, in pre-, mixed-, and mid-dilution HDF, higher substitution volumes are needed than in post-dilution HDF. Such higher volumes allow for increased plasma flow within the hemodiafilter and enhanced ultrafiltration flow in order to compensate for the lower solute concentrations and maintain the overall solute clearances throughout the dialysis session [32,33,34,35,36].

As described above, HDF requires the replacement of the fluid removed via ultrafiltration. In chronic kidney disease, this is mainly achieved via the use of online HDF [21]. During online HDF, the substitution fluid is not provided as a bagged, ready-made sterile fluid but is prepared during the treatment “online” from the dialysate fluid [21]. Cold sterilization of the substitution fluid is achieved via a two-stage ultrafiltration of the dialysate using sterilizing ultrafilters [21]. The use of specifically designed HDF machines and respective quality monitoring of the disinfection process combined with strict hygienic rules are mandatory [21]. Furthermore, HDF requires high-flux hemodiafilters with certain membrane characteristics and fiber geometry to achieve high convective clearance rates.

### 2.2. The Hemodiafilter

There are three requirements of a hemodiafilter that differentiate it from a standard HD dialyzer, which are as follows: (1) a sharp sieving coefficient curve to maximize the convective solute removal while retaining albumin; (2) high permeability to maximize the ultrafiltration volumes during HDF treatments; (3) a fiber geometry optimized to mitigate the effects of hemoconcentration [37].

First, to enable convective solute removal, particularly for middle-sized molecules, such as β2-microglobulin (~11.8 kDa), the membrane’s permeability for such solutes (measured through the associated sieving coefficient) must be sufficiently high. Membrane permeability is limited by the need to retain essential proteins such as albumin, as albumin loss may lead to the development of malnutrition [38]. Therefore, the ideal HDF membrane should have a steep sieving coefficient drop-off as follows: sieving coefficients of 1 for middle-sized molecules and sieving coefficients close to 0 for albumin (~66 kDa). This is summarized in the delicate balance expressed as the ratio between the molecular weight retention onset (MWRO) and the molecular weight cut-off (MWCO) that describe the best permeability slope.

**Figure 1:**
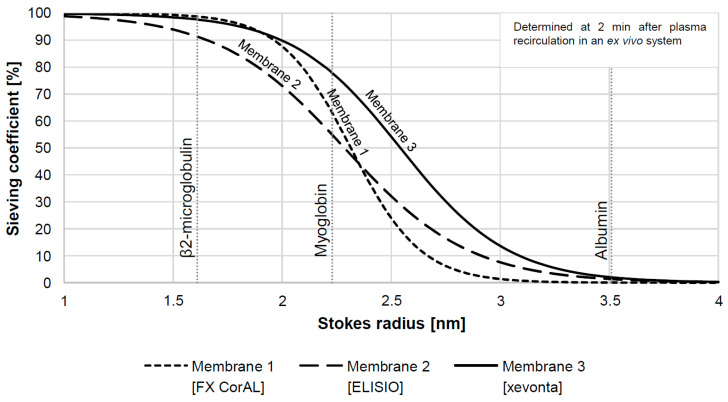
Schematic illustration of sieving coefficients as a function of Stokes radius for three different dialysis membranes. The sieving profiles were determined 2 min after plasma recirculation in an ex vivo system and differ in terms of retention properties across the range of solutes up to the size of albumin. Data were reanalyzed from recently published data [24] (n = 3 for each membrane; membrane 1: FX CorAL [Fresenius Medical Care]; membrane 2: ELISIO [Nipro]; membrane 3: xevonta [B. Braun]).

A schematic illustration of the sieving curve of three different dialysis membranes as a function of the solutes’ Stokes radius (corresponding to the molecular weight) is shown in Figure 1. Membranes 1 and 3 are essentially completely permeable for β2-microglobulin (~1.6 nm Stokes radius; ~11.8 kDa), while membrane 2 has an earlier MWRO and retains about 10% of the β2-microglobulin. In addition to the difference in MWRO, the slopes of the sieving curves also differ. The slope is an important determinant for the retention properties of larger proteins, such as albumin. Due to the high slope of membrane 1, this membrane has an earlier MWCO and features less permeability in the range of larger molecules as compared to membranes 2 and 3, leading to the advantage that its sieving coefficients for albumin (~3.5 nm Stokes radius; ~66 kDa) are about three times smaller than those of membranes 2 and 3 (~0.05% vs. ~1.5%). Thus, when selecting a membrane for HDF, membrane 1 would be the ideal candidate; the steeper slope between the MWRO and MWCO maximizes middle molecule removal while retaining albumin.

The second dialyzer requirement for optimal HDF performance is high permeability for plasma water to enable the large convective volumes desired, particularly during high-volume (HV) HDF treatments [10,12,13,39]. Because of this requirement, only high-flux dialyzers are used in (HV) HDF since the permeability of low-flux dialyzers is not sufficient.

Last, due to the enhanced ultrafiltration rates during HDF compared to HD treatments, the potential impact on rheology must be considered; the increase in blood viscosity is pronounced during HDF treatments, increasing the risk for fiber clogging [37,40]. To minimize these effects, it is advantageous for the hemodiafilter to favor low blood flow resistance (i.e., a low pressure drop across the blood compartment). This is typically achieved by utilizing hollow fiber membranes with a larger inner diameter (i.e., >200 µm). Additionally, it was shown that the improved flow dynamics in dialyzers with larger inner lumens in HDF treatments come with the advantage of increased convective volumes [41].

The currently marketed dialyzers and membranes for use in HDF essentially cover the full range of membrane materials available, such as common synthetic polymer membranes based on polysulfone, poly(aryl)ethersulfone, polyphenylene, acrylonitrile, polyester-polymer alloys, or polymethylmethacrylate, as well as membranes based on cellulose triacetate.

### 2.3. The Online HDF Machine

Besides the importance of high-flux hemodiafilters to achieve high substitution fluid volumes, the dialysis machine plays a substantial part in HDF treatments. High transmembrane pressure associated with large infusion volumes causes instable treatment conditions, multiple therapy interruptions, and cross-membrane protein losses [42]. Aiming for the best balance has led to a series of innovations in controlling the infusion rates during hemodiafiltrations.

In the early days of hemodiafiltration, the substitution fluids were provided in prefilled bags, limiting the infusion volumes to 5 or 10 L per session. When techniques became available to provide sterile and pyrogen-free fluids from online-supplied dialysate, larger infusion volumes were possible.

As a rule of thumb, the infusion rates were set at 25% of the blood flow to standardize the HDF treatment regimes. Larger infusion rates often resulted in treatment instabilities, leading to unplanned transitions from post-dilution to pre-dilution HDF. Adding patient-specific blood parameters, such as hematocrit and total protein concentration, together with dialyzer-specific data into the infusion rate calculation opened the door for the individualization of substitution volumes.

A milestone in HDF was the development of a dialyzer stress test during the dialysis. This pressure-based analysis of membrane stress due to ultrafiltration can be used to continuously tune the infusion rate. Different systems have been introduced, such as UltraControl™ (Gambro) or AutoSub plus™ (Fresenius Medical Care). Both control systems individualize and maximize the infusion rates by evaluating the pressure measurements at the dialyzer. Moreover, these innovations significantly decreased the complexity of performing online HDF while maximizing the intradialytic infusion rates in clinical routines [43,44]. 

UltraControl™ triggers an hourly scan of the ultrafiltration characteristics to determine the transmembrane pressure (TMP) set point, which is kept constant by adjusting the substitution rate during the next hour [45]. 

**Figure 2:**
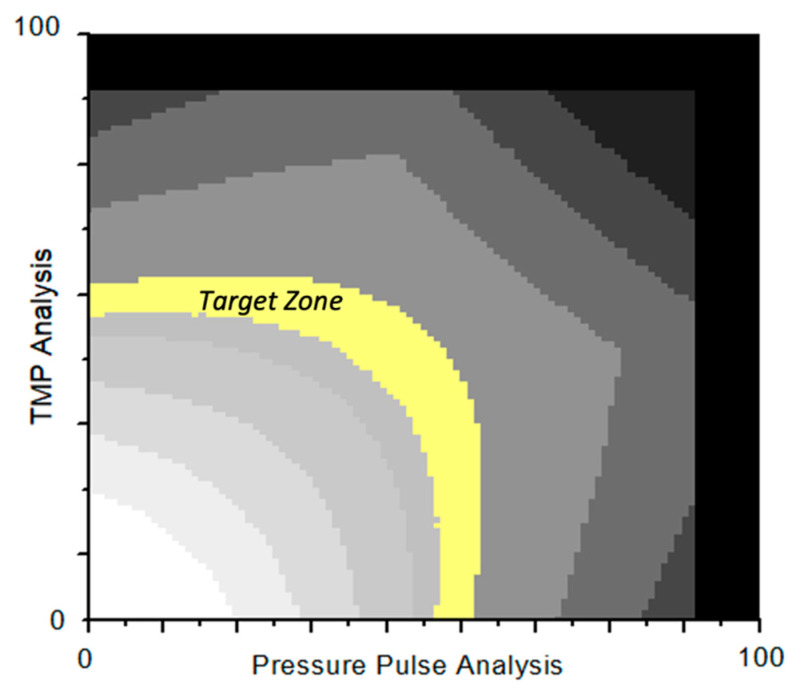
Substitution target matrix of AutoSub plus™. When the assessment is below the target zone, the substitution rate is increased to raise the stress in the membrane. When the assessment is above the target zone, the substitution rate is reduced to lower the stress in the membrane.

AutoSub plus™ also analyzes the transmembrane pressure but additionally includes information about the pressure modulations, which are generated by the peristaltic blood pump. These pressure modulations propagate through the capillaries of the dialyzer and are assessed downstream. The incremental attenuation of the pressure amplitude reflects an increase in the membrane stress. Both assessments, TMP and attenuation, are projected in a two-dimensional substitution target matrix (Figure 2). The designated target zone of the matrix opens a corridor, which is reached and maintained by feedback controlling the substitution rate.

AutoSub plus™ typically starts the substitution with a safe rate of 25% of the blood flow after the treatment has been commenced. Within a few minutes, the exchange rate is ramped up until the target stress level in the hemodiafilter is reached. The stress on the membrane is then continuously monitored to adjust the infusion rate. From a user perspective, hemodiafiltration can be as easy as hemodialysis, resulting in stable sessions with optimal substitution rates. The controller works in pre-dilution and post-dilution HDF with various dialyzer types without specific settings and permits significant increases in the ultrafiltration rate and volume. 

The ramping up process of the transmembrane pressure driven by the AutoSub plus™ software in the initial phase of the HDF session is a unique feature. By allowing time for the assembly of a protein layer at the beginning of the treatment, albumin loss may be significantly reduced. Indeed, it has to be remembered that almost 2/3 of the albumin loss in HDF occurs in the first hour; therefore, preventing albumin loss in such a way is an important and unique feature as far as high membrane stress is concerned.

Of note, besides UltraControl™ and AutoSub plus™, other feedback systems to maximize infusion rates are also available, such as the Surdial X’s Max Sub™ (Nipro) or the KUF Max™ (B.Braun) [46]. The Max Sub™ biofeedback procedure calculates the highest possible substitution rate individually for each patient based on a TMP control algorithm. The target TMP is set to about 250 mmHg. The substitution rate is adjusted to meet that value depending on the dialyzer membrane characteristic and blood composition of the patients, which individualizes the achieved exchange volume. The basis of the KUF Max™ biofeedback system is the definition of the systemic ultrafiltration coefficient K_UF,s_ as the ratio of the filtration rate Q_f_ and the TMP under operating conditions, or K_uf,s_ = Q_f_/TMP. In this case, the bent ultrafiltration (Q_f_–TMP) characteristics of the high-flux dialyzers lead to an increase in K_uf,s_ at low Q_f_ settings and a decrease in K_uf,s_ at higher Q_f_ settings. The maximum systemic K_uf,s_ based on this definition is the KUF Max. It is characterized by the onset of a strong increase in oncotic pressure, indicating the formation of a concentration polarization layer of non-permeable plasma proteins on the membrane surface. The KUF Max is determined at the beginning of the treatment and used as a target setting for the treatment. It delivers the filtration rate Q_f_ with the lowest TMP/Q_f_ ratio in contrast to the highest achievable filtration rate, which is the aim of the UltraControl™, AutoSub-Plus™, and Max Sub™ concepts.

High filtration rates are prone to hemoconcentration, clotting, and loss of dialyzer surface area. To circumvent any troubles during the treatments, biofeedback algorithms must be designed to detect the onset of adverse developments and incorporate system dynamics to facilitate an appropriate response. Continuous monitoring is mandatory to enable a prompt automated intervention when necessary.

### 2.4. Impact of Protein Fouling of the Membrane on Substitution Volume

As shown above, membrane permeability and the TMP between the blood and dialysate compartments are important determinants of the substitution volume that is achieved during an HDF treatment. The contact of human blood with the artificial membrane surface leads to the adsorption of plasma proteins and the formation of a secondary membrane [23,24,25,26,27,28,29,47]. This secondary membrane, which is composed of plasma proteins, is an additional barrier to water and uremic toxins and reduces the initial permeability of the hemodiafilter, which in turn requires a higher TMP to achieve the same ultrafiltration flow.

**Figure 3:**
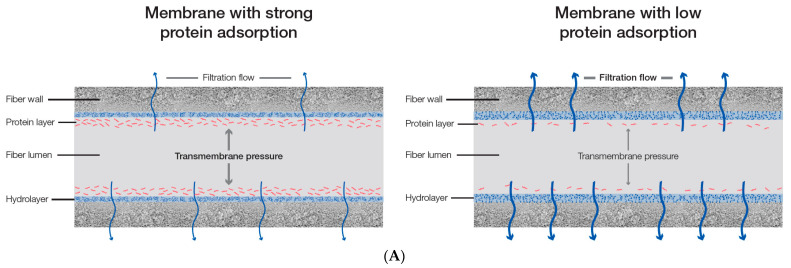

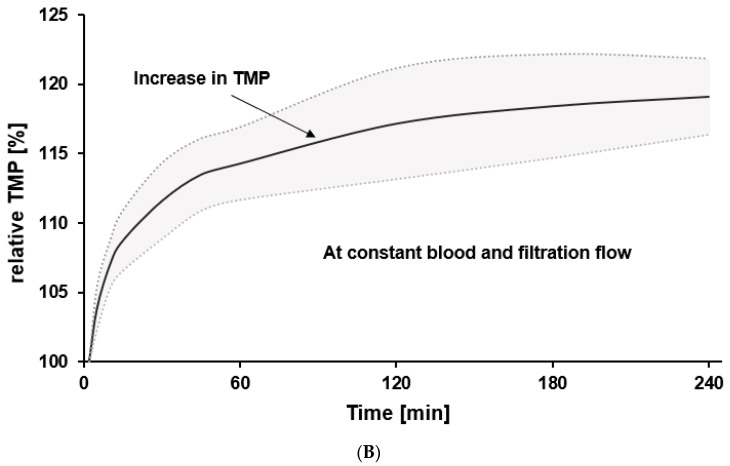
Impact of protein adsorption on the hydraulic characteristics of the membrane. (**A**) The membrane with strong protein adsorption (left) provides higher mass transfer resistance than the membrane with low protein adsorption (right), leading to a lower filtration flow rate and/or a higher transmembrane pressure (TMP). (**B**) At constant blood and filtration flows, the build-up of a secondary membrane leads to an increase in the TMP over the treatment time. Data were reanalyzed from a recently published study [24]. The solid line represents the mean value of three different dialyzers (FX CorAL (Fresenius Medical Care), ELISIO (Nipro), and xevonta (B. Braun); n = 3 for each dialyzer type), whereas the dotted line represents the standard deviations. Data were measured at the following time points: 2, 5, 10, 15, 30, 45, 60, 120, 180, and 240 min.

Decreased membrane permeability due to protein fouling occurs in both HD and HDF treatments, but its role is significantly more important during HDF [25,48,49]. Given the relatively low ultrafiltration rate during an HD treatment, the transportation of plasma proteins to the inner membrane surface is slower than during HDF treatments [48,50]. Because of the faster transport rate of blood proteins to the membrane surface during HDF, the secondary membrane-induced mass transfer resistance tends to be more pronounced. The additional resistance from the secondary membrane affects the HDF treatment by reducing the removal capacity of the solutes and the hydraulic permeability of the membrane [24,25,26,27,28,29,47,48,49,50,51]. Thus, when blood or plasma is circulated through a dialyzer at a constant blood flow and TMP, this leads to a reduction in the filtration rate over time. We recently investigated this phenomenon in a recirculation model with human plasma over 4 h [24]. In this setup, the blood and filtrate flows were kept constant during the recirculation experiment with three different synthetic dialyzers. We observed a continuous increase in the TMP, which was especially pronounced in those dialyzers that showed strong protein adsorption. The average increase in the TMP was about 20% throughout the 4-hour experimental period. Here, the strongest TMP increase was especially seen at the beginning of the recirculation (in the first 30 min), given that in this time period, the secondary membrane is mainly formed [23,24,52]. A schematic description of the impact of protein adsorption on the hydraulic characteristics of the hemodiafilter membrane is presented in Figure 3.

**Figure 4:**
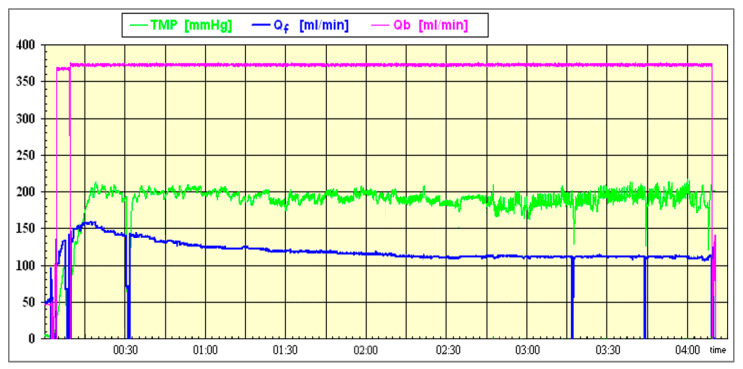
Post-dilution online HDF treatment with control of substitution by AutoSub plus™; the dialyzer used was the FX60 (Fresenius Medical Care).

The reduced permeability of the membrane is evident to the machine during the dialyzer stress tests performed during the treatment. As a mitigation for the increased TMP caused by the protein adsorption, the substitution rate is automatically reduced. This effect is particularly conspicuous in the first half an hour after the target stress level has been reached (Figure 4). The reduction in the substitution rate is necessary to keep the treatment stable. Inevitable alarms and treatment interruptions are consequently avoided for nurses and patients. Notably, this reduction in the infusion rates finally leads to smaller substitution volumes. This can be a hindrance to achieving the maximum convective volumes.

The impact of protein adsorption on the membrane is, therefore, two-fold in the following context: first, it reduces the achievable exchange volume, and, second, it reduces the sieving coefficients of larger middle molecules. Both effects are multiplicative in the removal of these substances.

Thus, to achieve good hydraulic characteristics during an HDF treatment, a hemodiafilter should minimize the protein adsorption on its membrane surface. For synthetic dialysis membranes, clinical and experimental studies have demonstrated that hydrophilic modification of the membrane surface reduced the protein adsorption and led to performance stability during treatments [23,24,52,53,54]. Hydrophilic modification of synthetic dialysis membranes is commonly achieved by blending the hydrophobic membrane polymers with polyvinylpyrrolidone (PVP), which is an inert and highly hydrophilic agent and has been shown to reduce protein adsorption via the repulsive hydration force of the formed water layer [55,56,57,58,59,60]. We recently compared the shift in molecular weight retention curves during in vitro plasma recirculation among three dialyzers with different PVP contents on the blood-side surface of their membranes [24,52,61]. Here, the dialyzer with the highest PVP content on the blood-side surface of the membrane showed the lowest protein adsorption and induced the lowest shift in the molecular weight retention curves, indicating the strongest stability in performance over treatment time [24,52,61]. This was confirmed in a randomized controlled trial with 52 hemodialysis patients treated with three synthetic dialyzers in the post-dilution HDF mode. The results of this study showed that the dialyzer with the highest PVP content and the lowest protein adsorption was superior in the removal of β2-microglobulin as compared to the other two dialyzers [53].

Besides PVP as a common hydrophilic agent in hemodialysis membranes, some other hydrophilic co-polymers also exist in synthetic dialysis membranes, such as the hydrophilic sodium methallylsulfonate as a co-polymer of the hydrophobic polyacrylonitrile in AN69^®^ membranes. Moreover, the hydrophobic polyacrylonitrile can also be made hydrophilic by the co-polymerization with methylmethacrylate and the addition of acrylic acid in the case of PAN membranes. To the best of our knowledge, no data exist regarding the characterization of such other hydrophilic modifications with regards to the impact on maintaining filtration characteristics due to lower protein fouling during HDF treatments.

To maximize the substitution volume that is achieved during an HDF treatment, special consideration needs to be given to the technical components of the HDF system. In this context, the operating conditions (i.e., blood flow, anticoagulation, and transmembrane pressure management) and the patient’s vascular access and hemorheological characteristics must be considered when prescribing HDF and assessing its performance.

## 3. Medical Insights of HDF

First, the present section describes the clinical relevance of good treatment options for the highly comorbid ESKD patient population. Next, the differences in treatment modalities with regards to treatment efficiency, such as performance, are discussed. Finally, this section summarizes the clinical evidence regarding hard clinical outcomes in HDF as compared to other treatment modalities and discusses the potential mechanisms of the beneficial effects of HDF on morbidity and mortality.

### 3.1. Necessity of Advanced Treatment Options for ESKD Patients to Improve Outcomes

Advances in technical and medical treatment options are of paramount importance for patients with ESKD based on their high morbidity and mortality rates [1,2,3,4,5]. In a recent study with ESKD patients treated with HDF, more than 90% suffered from metabolism and nutrition disorders, including type 2 diabetes mellitus [53]. Vascular disorders, such as hypertension, were reported for approximately 85% of patients, and cardiac disorders/coronary artery disease were reported for approximately 65% of patients. These comorbidities are both a sequel and an origin of ESKD. According to investigators, hypertensive and large vessel diseases (48%) and diabetes mellitus (21%) are the two leading root causes of ESKD.

In this context, cardiovascular complications are a leading cause of death in ESKD patients [3,6,62,63,64,65]. Male patients with ESKD on hemodialysis have an overall incidence of myocardial infarction of approximately 2.13 per 100 patient-years [66], which is approximately 3.5 times the risk of a comparable male population of >65 years old and not on hemodialysis [67]. The overall mortality rate in this population is approximately 6 times higher than in the general population [68]. This risk is also driven by vascular calcification caused by hypercalcemia and a dysregulation of the parathyroid hormone [69,70]. Moreover, diabetes mellitus and protein–energy wasting (PEW) are major problems among dialysis patients and major predictors of morbidity and mortality [71,72,73,74,75]. One major component of PEW is anorexia, which affects up to 50% of ESKD patients [76]. Anorexia may be caused by underdialysis, which, in turn, affects taste. A randomized study of 30 patients showed that an adaptation of the dialysis dose from a low initial Kt/V of 0.82 ± 0.19 to 1.32 ± 0.21 increased protein uptake (estimated by the protein catabolic rate, or PCR) by 26%, whereas the PCR remained constant in the control group with the unchanged dialysis procedure [77]. It is, thus, important to achieve the recommended dialysis target doses to avoid the PEW syndrome, e.g., by using highly efficient dialysis modalities. In addition to protein intake, protein loss should be considered, as the sieving properties of dialyzers may contribute to the PEW syndrome if they leak elevated amounts of albumin [23,53]. Thus, efficient dialysis may alleviate comorbidities associated with negative clinical outcomes in ESKD patients. This should include the elimination of middle- and large-sized uremic toxins, such as β2-microglobulin or inflammatory cytokines, and prevent the loss of essential proteins, such as albumin.

### 3.2. HDF vs. Other Modalities: Impact on Performances as Short-Term Surrogate Marker of Efficiency

HDF has become the renal replacement therapy of choice in Europe and in Asian countries due to its superior performance vs. HD, i.e., the clearance and removal of uremic toxins, improved intradialytic hemodynamic stability, including fewer periods with cardiac and vascular stresses, and reduced inflammation [13,78,79,80,81]. The present section compares the performance characteristics of HDF with those of other treatment modalities.

Superior performance originates from combining the physical principles of diffusion known from HD and convection [7,10,11]. Online HDF outperforms low-flux HD in the removal of middle molecular weight uremic toxins, such as β2-microglobulin, myoglobin, and leptin [15,82,83,84]. For β2-microglobulin, however, high-flux HD may achieve comparable results as online HDF [12,14]. Furthermore, HDx (expanded HD) with medium cut-off (MCO) membranes and forced internal filtration by design has been described as an additional treatment modality. MCO membranes may achieve nominally similar solute clearances, and they possess good removal capacities for molecules up to approximately 50 kDa, with an albumin loss of below 5 g per HD session [28,85]. In a large survey among 71 Italian nephrologists, four questions were dedicated to MCO membranes [86]. Using the Delphi method of sequential questionnaires and defining a consensus as a level of agreement of ≥66%, the experts agreed that MCO membranes were associated with reduced systemic inflammation, improvement of dialysis-related anemia, better clearance for middle-to-high molecular weight uremic toxins, and improved treatment hemodynamics. However, the experts did not see a consistent association between MCO membranes and reduced mortality, especially from cardiovascular causes (a borderline positive consensus of 66%).

To describe the performance and safety of different dialysis modalities, Maduell and Broseta performed a selective review of the published clinical study data [87]. As an indicator of performance, they defined a global removal score as the average removal rates of β2-microglobulin, myoglobin, prolactin, α1-microglobulin, and α1-acid glycoprotein minus the removal rate for albumin. Post-dilution HDF possessed the highest mean removal score, followed by HDx, which was superior to pre-dilution HDF and high-flux HD. Low-flux HD presented the lowest global removal score, which was less than half that of post-dilution HDF. When analyzing the removal rates depending on blood flow (Qb) and substitution volume, post-dilution HDF proved superior to HDx, even with low Qb and a substitution volume not smaller than 17–18 L per session [88,89,90]. Regarding safety, the authors stressed that MCO membranes must only be used in HDx and not in HDF. Here, >20 g of albumin could be lost per session, whereas dialyzers dedicated to HDF may sieve as little as <2 g [53,91]. Furthermore, in the CARTOON trial, a randomized trial comparing HDx and post-dilution HDF, the coronary calcium scores, a surrogate for cardiovascular outcomes, remained stable in the HDF group and deteriorated significantly under HDx [92,93].

Good dialysis performance could also be linked to the improvement of other complications in dialysis patients. A cross-sectional study among 82 non-diabetic dialysis patients compared the effects of HD and HDF on the insulin resistance index [94]. The study found that insulin resistance was significantly correlated with the β2-microglobulin reduction rate and HDF was associated with lower insulin resistance compared to HD. This indicates that HDF, which is generally superior in the β2-microglobulin reduction rate to standard HD, might preserve insulin sensitivity in non-diabetic patients on renal replacement therapies or improve insulin resistance in diabetic patients. Furthermore, HDF is associated with less inflammation compared to standard HD, as shown by the lower levels of CRP, interleukin 6 (IL-6), and homocysteine. This could be linked to the better elimination of some inflammatory compounds, such as advanced glycosylation end products (AGEs), which accumulate in patients with ESKD and activate monocytes to release IL-6, TNF-α, and interferon-γ [82,95,96,97,98].

In this context, it is important to note that for efficient HDF treatments, suitable high-flux dialyzers are essential to allow for strong performance throughout the complete treatment time [23,24]. Several randomized controlled trials have been performed to compare the performance of different dialyzers among post-dilution HDF [53,54,99]. The trials found higher mean β2-microglobulin reduction rates for the dialyzers with synthetic membranes (>67% over a 4 h session) compared to dialyzers with cellulose triacetate-based membranes (51%). The dialyzers with polysulfone-based membranes achieved β2-microglobulin reduction rates of >70% in all of the trials. Here, the dialyzer possessing the most hydrophilic membrane and the lowest protein fouling showed the best removal of middle-sized molecules.

A recent cross-over study in ten stable HD patients by Vanommeslaeghe et al. compared the membrane fiber patency and performance in post-dilution HDF between a cellulose-based asymmetric triacetate (ATA) dialyzer (Solacea 19H) and a polysulfone dialyzer (FX CorDiax 800) [100]. The ATA dialyzer maintained open fibers over the dialysis sessions, whereas the polysulfone dialyzer showed a declining patency towards the end of the dialysis. The performance was generally in line with the fiber patency. These results appear to contradict the trials cited above and the laboratory data [23,52], where polysulfone dialyzers were superior to cellulose-based dialyzers. However, the study by Vanommeslaeghe et al. submitted the dialyzers to a fiber-blocking stress test that is not relevant for current clinical practices in that the dose of anticoagulation was reduced to one-fourth of the regular dose. Furthermore, the polysulfone dialyzer was a predecessor to the current model with improved hydrophilicity and fiber patency [23,52].

### 3.3. Impact on Morbidity and Mortality as Hard Clinical Endpoint to Support Larger Use of HDF

The most current clinical evidence suggests that HDF offers better clinical outcomes regarding the survival rate of dialysis patients as compared to standard HD, especially when delivered in high-volume mode. This section summarizes the clinical evidence with regards to morbidity and mortality. 

As discussed above, the addition of convection to the basic mechanism of HD (diffusion) improves the clearance of middle molecular weight solutes during online HDF. This additional correction of the uremic environment in HDF is associated with decreased cardiovascular damage and, subsequently, lower cardiovascular morbidity and mortality [101]. This hypothesis was tested in four large randomized controlled trials (RCTs), all of which were performed in Europe. However, none of the studies provided undisputable results to the basic question of whether HDF is superior or not [12,14,15,102,103]. 

In an individual patient data meta-analysis (IPD-MA), the European pooling project combined the four RCTs (N = 2793 patients) that compared HDF (N = 1400, post-dilution mode) to standard HD (N = 1393) on clinical outcomes [13] (Table 1). This analysis found that there was a 14% reduction in all-cause mortality and a 23% reduction in cardiovascular mortality when treated on HDF as compared to standard HD, which the authors classified as a substantial effect. The other causes of death, including sudden death and non-cardiac events, such as fatal infections and malignancies, were equally distributed between the HD and HDF groups. An interesting aspect of all four trials was that the actually delivered dosage of convective volume showed a considerable non-intended range caused by variations in everyday clinical practice. This fact and the patients’ given height and weight made it possible to perform post-hoc analyses on the possible associations between the convective volume standardized to body surface area (BSA) and survival outcomes. Therefore, the group of HDF patients was divided into the following three tertiles by delivered convective volume: low volume of <19 L, middle volume of 19–23 L, and high volume of >23 L per session. With respect to all-cause mortality, there was evidence of a dose–response relation (Table 2). The highest delivered BSA-adjusted volume (>23 L per 1.73 m^2^ BSA per session) was associated with a 22% reduction in all-cause mortality and a reduction of 31% in cardiovascular mortality after an adjustment for age, gender, albumin, creatinine, history of CV diseases, and history of diabetes. These 23 L were based on the lower limit of the highest tertile in this meta-analysis. Based on these results, many subsequent publications have recommended a minimum convection volume of 23 L/1.73 m^2^ BSA per session for HDF treatments [13,78]). Furthermore, scaling of the ultrafiltration volume to BSA allows for the adjustment of the dialytic convective dose to a patient’s metabolic needs and the comparison of populations with various anthropometric profiles (i.e., European, Asian, or American populations).

Bernard Canaud and co-workers reached a comparable conclusion in an observational study (N = 2293) [39]. Here, the recommended convection volume was approximately 70 L per week, i.e., approximately 23 L per session, and, again, the data suggested a dose–response relation between the convective volume and the relative survival rate. 

Another large observational study from Japan, among 5000 pairs of patients treated with HD or pre-dilution online HDF (the usual HDF mode in Japan), investigated the association of HD versus HDF (low and high volume) with all-cause and cardiovascular mortality [33]. In the pre-dilution mode, the substitution volume usually doubles. Thus, the high-volume HDF in the study was at about 50 L per session, and the low-volume HDF was at 25 L per session. Based on this definition, the 12-month all-cause mortality in the high-volume HDF group was significantly lower than that in the low-volume HDF group. Interestingly, when comparing the HD and high-volume HDF, the survival curves for all-cause mortality and cardiovascular mortality diverged very fast, which suggested a rapid effect. 

Recently, the French Renal Epidemiology and Information Network Registry, which was started in 2002 with the aim to generate real-world evidence data for HDF and HD [104], also confirmed in a large observational study (REIN Registry) the superiority of HDF vs. HD with regards to all-cause and cardiovascular mortality [105].

In contrast, a French RCT [103], which focused on 381 elderly patients (above 65 years of age), and a newer analysis of Dialysis Outcomes and Practice Study (DOPPS) data of 8567 patients [106] did not find a significant difference in either all-cause or cardiovascular mortality between HDF and HD or between HDF patients with convective volumes below versus above 20 L per session.

Using a more detailed analysis of 2793 patients from the European pooling project, the authors investigated whether the benefits of HDF regarding cardiovascular mortality depend on the type of cardiovascular disease, i.e., cardiac cardiovascular disease, non-cardiac cardiovascular disease, or unclassified cardiovascular disease [13]. This analysis showed that the reduction in cardiovascular mortality in the HDF mode was solely explained by the cardiac part of cardiovascular mortality. 

Thus, most studies found HDF, at least when delivered in high-volume mode, to be associated with a reduction in all-cause mortality. This was mainly explained by a reduction in cardiovascular and, more specifically, cardiac mortality [107,108]. 

### 3.4. Mechanisms for Beneficial Effects of HDF

This evidence on the beneficial effects of HDF raises a question regarding the possible mechanisms of the observed effects on improved survival reported for HDF. In a recent review article, two groups of associated effects—direct and indirect effects—were discussed with regards to cardiovascular function and structure [7]. Regarding the direct factors, data from observational and interventional multicenter studies show a reduction in the frequency of intradialytic hypotension episodes in convective therapies, such as HDF or HF vs. HD, a better hemodynamic stability [109,110,111,112,113], and an improvement in cardiac remodeling during HDF treatments [114,115]. A better hemodynamic stability appears not to be related to the better Na^+^ balance achievable by HDF vs. high-flux HD [116]. Other direct effects may include the following: a reduction in chronic inflammatory states [98,114,117,118], oxidative stress [118,119,120], or an improvement of endothelial function and cardiovascular stiffness [16,121,122]. Furthermore, the direct effects can also be a reduction in the progression of atherosclerosis [32,118], sympathetic tone (nerve) activity [123], arrhythmogenicity, or the circulation of cardiotoxic uremic toxins [16,124]. Importantly, these direct effects may affect each other. Indirect effects in this context comprise a better correction of anemia [125,126], a better nutritional state [127,128], an improvement in physical activity [129,130], a better quality of life [131,132,133], and maintenance of residual kidney function [134,135]. It is highly difficult or basically impossible to separate these effects and identify a common underlying effect. It is most likely a complex of various interrelated effects that overall result in a beneficial effect on clinical endpoints. In this context, patient-related factors precluding a sufficiently high convective volume exchange may play a role. A retrospective study performed by Davenport identified low post-sessional intracellular water, low serum albumin, diabetes mellitus, and higher co-morbidity as indicators for low convective volumes. As these factors are not easily remediable, the patients affected may not be able to achieve the higher convection volume reported to be associated with improved patient survival [136].

In summary, the effects on the removal of middle molecules and large soluble molecules, inflammation, intradialytic morbidity, endothelial function, blood pressure regulation, and oxidative stress are the factors with the clearest evidence in the literature at present [7,137].

## 4. Conclusions and Future Directions

In summary, both technical and medical aspects are important to consider for the treatment of ESKD patients with HDF. Both the membrane and the machine may impact the achievement of treatment goals, especially regarding convection volume targets. Importantly, lower protein adsorption to the membrane may be beneficial for the filtration characteristics of the dialyzer and may help to achieve high convection volumes during HDF. The development of membranes with lower protein adsorption may help to better achieve these treatment goals. Efficient dialysis treatment options are of paramount importance for these highly comorbid patients, and the latest clinical studies point towards better outcomes (mortality and cardiovascular disease) for those patients treated with high-volume HDF. Apart from the technical aspects delineated in this review, it must be highlighted that the implementation of best clinical practices is also of crucial importance, since it has been shown that most of the differences observed in recent RCT trials may be likely explained by practice variations. 

Regarding future clinical studies, more focus should also be given to other outcome measures, including quality of life or patient-related outcome measures, beside the reduction in all-cause and cardiovascular-related mortality [17,18,138,139,140]. Any meaningful differences in patient-reported outcome(s) could already be a strong selection criterion for a respective treatment option. Differences in patient-reported outcomes certainly need a different understanding of the underlying mechanisms as compared to those mechanisms identified for hard clinical outcomes. A better removal of certain solutes and improvements in biological and physiological processes may lead to a reduction in symptoms, improvements in body functions and general health perception, and, finally, better health-related quality of life (HRQL). These biological and physiological mechanisms that have an effect on HRQL clearly need more attention in the future.

Evidence regarding the superiority of HDF vs. HD with regard to patient-related outcomes is still scarce, and even in terms of mortality, the debate is still ongoing [138,139,141]. Presently, two large trials (CONVINCE and H4RT) are being performed in Europe and may close these gaps as they address the question of whether high-dose HDF (a convection volume of >20 L) possesses any benefits for the patients versus high-flux HD [17,18,140]. CONVINCE is an international, multicenter, controlled, and randomized trial that started in 2018 [17,140]. H4RT is a British trial with a comparable design that started in 2017 [18]. Both of them examine all-cause mortality as their primary objective. However, to capture the patients’ perspectives and compare these between the two treatment groups, the trials’ secondary objectives include patient-related outcomes, such as quality of life. Furthermore, both trials address the economic aspects of renal replacement therapy. Thus, presently, approximately 3400 patients from more than 100 dialysis centers in various countries in Europe are being included in these trials. The study groups aim to present their final results in 2023. 

In conclusion, there is more and more evidence that HDF delivered at a high dosage improves performance as compared to standard HD treatments. Furthermore, HDF is associated with better clinical outcomes for traditional or hard endpoints, such as all-cause and cardiovascular mortality. Besides patient characteristics, the technical aspects, including the dialyzer and the machine, are the key elements in providing efficient HDF treatments for ESKD patients. In the near future, large-scale controlled trials, such as CONVINCE and H4RT, will hopefully deliver definite proof that HDF is superior to HD when looking at the hard endpoints. An additional and important feature of these trials is to extensively and comprehensively analyze patient-reported outcomes and economic questions, which may finally lead to the optimization of renal replacement therapy for ESKD patients.

## Figures and Tables

**Table 1:** European pooling data meta-analysis study [13].

Reference	Study Name	Number Patients	Countries	Study Design	Comparison	Mortality Endpoints
Grooteman et al., 2012 [15]	CONTRAST	714	Netherlands, Canada, Norway	Multicenter randomized controlled clinical trial	Online post-dilution HDF versus low-flux HD	All cause-mortality, Cardiovascular vs. non-cardiovascular mortality
Maduell et al., 2013 [12]	ESHOL	906	Spain	Multicenter randomized controlled clinical trial	Online post-dilution HDF versus high-flux HD	All cause-mortality, Cardiovascular vs. non-cardiovascular mortality
Ok et al., 2013 [14]	Turkish HDF study	782	Turkey	Multicenter randomized controlled clinical trial	Online post-dilution HDF versus high-flux HD	All cause-mortality, Cardiovascular vs. non-cardiovascular mortality
Canaud et al., 2004 [102]	French HDF study	391	France	Multicenter randomized controlled clinical trial	Online HDF versus high-flux HD	All cause-mortality, Cardiovascular vs. non-cardiovascular mortality

**Table 2:** Hazard ratio (HR) for mortality outcomes by delivered convection volume per session for online HDF compared to standard HD [13].

Endpoint	HR (95% Confidence Interval)		
	Low Volume < 19 L	Mid Volume 19–23 L	High Volume > 23 L
All-cause mortality	0.83 (0.66–1.03)	0.93 (0.75–1.16)	0.78 (0.62–0.98)
Cardiovascular mortality	0.92 (0.65–1.30)	0.71 (0.41–1.03)	0.69 (0.47–1.00)

The HR values are adjusted for age, gender, albumin, creatinine, history of CV diseases, and history of diabetes.

## Data Availability

Not applicable.
